# Determination of the ideal plate for medial femoral condyle fracture fixation: an anatomical fit and biomechanical study

**DOI:** 10.1186/s12891-024-07374-5

**Published:** 2024-04-16

**Authors:** Felix Leung, Christian Xinshuo Fang, Colin Shing Yat Yung, Frankie Ka Li Leung

**Affiliations:** 1 Department of Orthopaedics and Traumatology, Queen Mary Hospital, 5/F, Professorial Block, 102 Pokfulam Road, Pokfulam, Hong Kong, China; 2 Department of Orthopaedics and Traumatology, The University of Hong Kong, Hong Kong, China

**Keywords:** Medial, Femoral, Condyle, Fracture, Fixation, Anatomical, Biomechanical

## Abstract

**Background:**

The aim of this study is to determine the best plate to use as a substitute to fix a medial femoral condyle fracture.

**Materials and methods:**

The first part is to measure the best fit between several anatomical plates including the Proximal Tibia Anterolateral Plate (PT AL LCP), the Proximal Tibia Medial Plate (PT M LCP), the Distal Tibia Medial Locking Plate (DT M LCP) and the Proximal Humerus (PHILOS) plate against 28 freshly embalmed cadaveric distal femurs. Measurements such as plate offset and number of screws in the condyle and shaft shall be obtained. The subsequent part is to determine the compressive force at which the plate fails. After creating an iatrogenic medial condyle fracture, the cadavers will be fixed with the two plates with the best anatomical fit and subjected to a compression force using a hydraulic press.

**Results:**

The PT AL LCP offered the best anatomical fit whereas the PHILOS plate offered the maximal number of screws inserted. The force required to create 2 mm of fracture displacement between the two is not statistically significant (LCP 889 N, PHILOS 947 N, *p* = 0.39). The PT AL LCP can withstand a larger fracture displacement than the PHILOS (LCP 24.4 mm, PHILOS 17.4 mm, *p* = 0.004).

**Discussion and conclusion:**

Both the PT AL LCP and the PHILOS remain good options in fixing a medial femoral condyle fracture. Between the two, we would recommend the PT AL LCP as the slightly superior option.

**Supplementary Information:**

The online version contains supplementary material available at 10.1186/s12891-024-07374-5.

## Background

Despite advances in implant design, distal femoral condyle fractures remain to be a challenging fracture to manage; these fractures are often intra-articular, and patients are easily prone to functional limitations in the setting of complications. The commonly accepted modes of definitive fixation include a lateral femoral condyle plate or dual plating (lateral and medial). Though there are several commercially available anatomically designed plates for the lateral femoral condyle, there remains to be no anatomical plate specifically designed for the medial femoral condyle [1]. Historically, many other plates have been used to substitute a medial femoral condyle plate, including the proximal tibia LCP or the TomoFix Medial Distal Femur, but the best plate is yet to be determined [1–4]. Additional factors compromising fixation include osteoporosis [5], compromised mechanical stiffness when using annealed “reconstruction” plates [6] and fracture comminution.

One of the more popular substitutes for a medial femoral condyle plate is to use the anterolateral proximal tibial plate of the ipsilateral leg placed in a reverse position [3]. Silva et al. recently proposed a more innovative idea by fixing the medial femoral condyle with a calcaneal plate [4]. Upadhyay recently published a study where he compared 18 different pre-contoured anatomical plates to find the plate best fitting the medial femoral condyle [1]. He concluded that the anterolateral proximal tibia plate and the proximal humerus (PHILOS) plate both have the best fit along with at least 6 screws inserted with no notch penetrance. To date, there has been no further evidence on to which plate is superior. The aim of this study is to determine which is the best plate, defined as the plate with the best anatomical fit and best biomechanical stability. Our null hypothesis is that there are no statistical differences amongst all plates.

## Methods

### Study design

The study was approved by the institutional review board of our hospital. Twenty-eight freshly embalmed cadaveric distal femurs (14 left distal femurs and 14 right distal femurs) were used. The age of death ranged from 66 to 98 years old (mean 83.7) and all were female. The soft tissues were removed.

### Anatomical fit

The study was divided into two parts. The first part consisted of measuring the best anatomical fit between several anatomical plates including the 3.5 mm LCP® Proximal Tibia Plate (PT AL LCP), the 3.5 mm LCP® Medial Proximal Tibia Plate (PT M LCP), the 3.5 mm LCP® Medial Distal Tibia Plate (DT M LCP) and the Proximal Humerus Interlocking System (PHILOS) plate when fixed against the medial side of the distal femur. 28 freshly embalmed cadaveric distal femurs were used (14 left distal femurs and 14 right distal femurs). All plates were from DePuy Synthes.

**Fig. 1:**
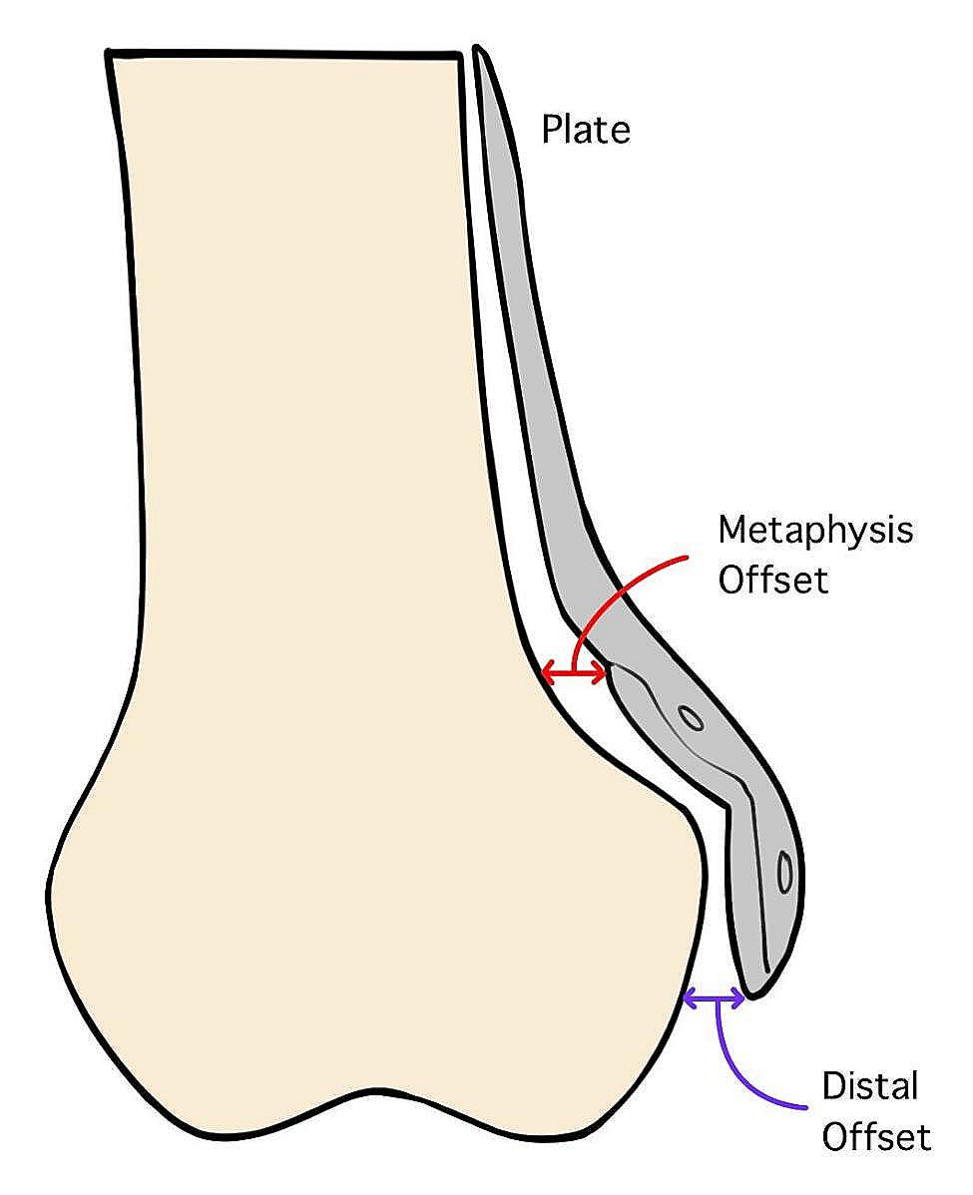
Parameters measured for determining anatomical fit (AP view)

**Fig. 2:**
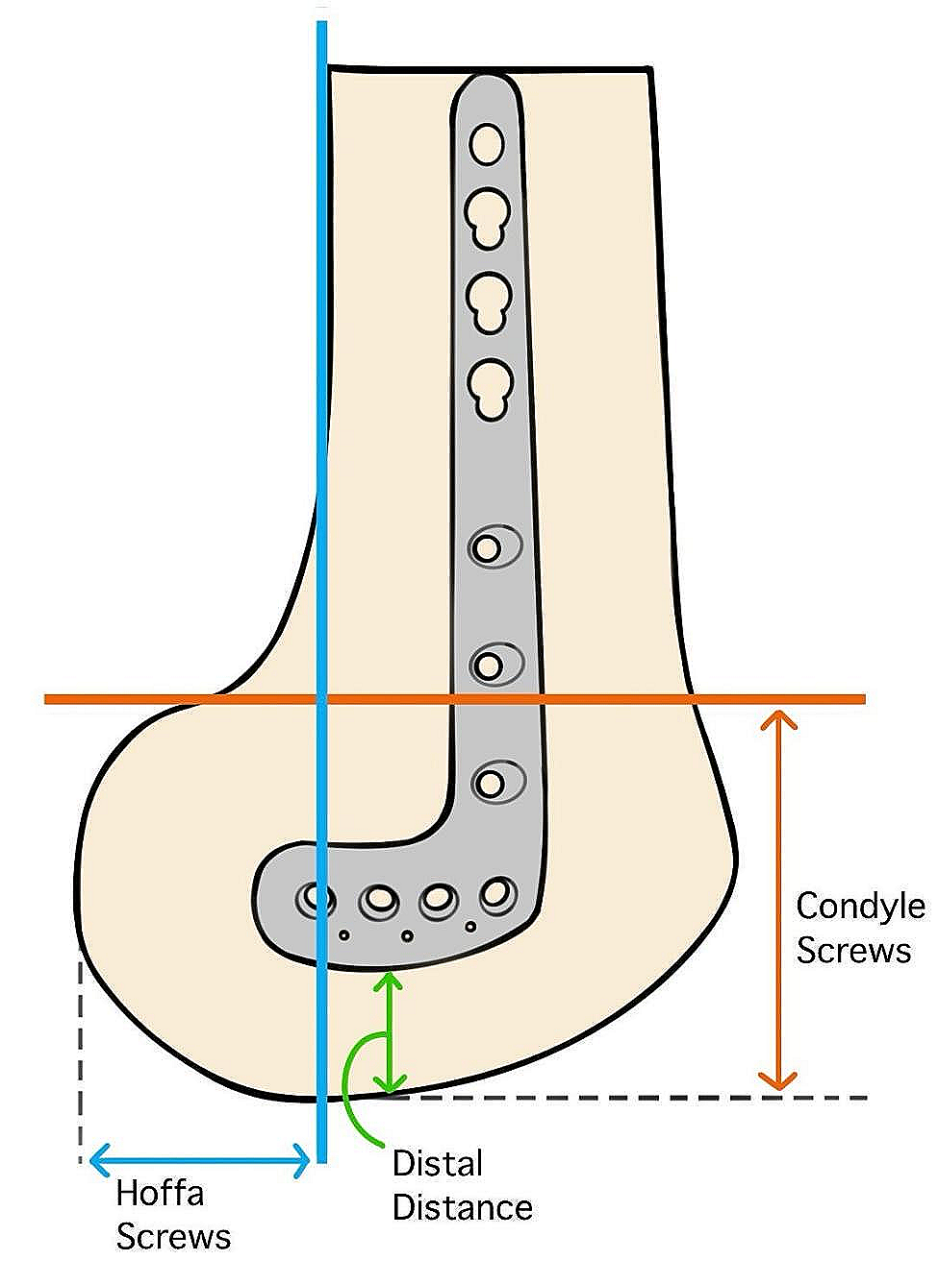
Parameters measured for determining anatomical fit (lateral view)

Plate fitting assumes that no contouring of the implant is needed, and the implant should fit well on the surface of the medial condyle with sufficient distal screw purchase. Here, the primary outcomes were defined by the amount of metaphysis offset (AP view), the amount of distal offset (AP view), the distal distance (lateral view), the number of screws in the Hoffa fragment, the number of screws in the condyle and the number of screws penetrating the joint. Figures 1 and 2 depict how we measured our parameters. The amount of metaphysis offset is defined as the largest distance between the plate and the metaphysis. The amount of distal offset was defined as the largest distance between the distal part of the plate with the epiphysis. The distal distance is defined as the distance between the edge of the plate with the edge of the femoral cortex and the joint line and is measured in the lateral view. The number of Hoffa screws is defined as the number of screws being completely buried posterior to a line drawn from the posterior femur cortex. The number of condyle screws is defined as the number of screws being completely distal to a line perpendicular to the posterior femur line at the level of the confluence between the metaphysis and the femoral condyles offset of the posterior condyle.

In each primary outcome, we ranked the 4 plates from first to last. The two plates that were most ranked first were then used in the second part of the study.

### Biomechanical study

The second part of the study was to determine the force displacement characteristics of the two constructs. A fracture model consisting of a medial condyle fracture was created in all 28 specimens. An osteotomy in the sagittal plane was made from the mid-point between the articulating condyles to the confluence of the medial condyle with the metaphysis. The fracture was then anatomically reduced and fixed by one of the two plates chosen from the first part of the study. No contouring was performed for each plate such that its native best position was used for plate placement. Each plate was fixed with four distal locking screws and three proximal locking screws. Only fixed angled 3.5 mm locking screws were used. Compression cortical screws were not used to ensure no change in plate contouring and to ensure consistency between specimens.

**Fig. 3:**
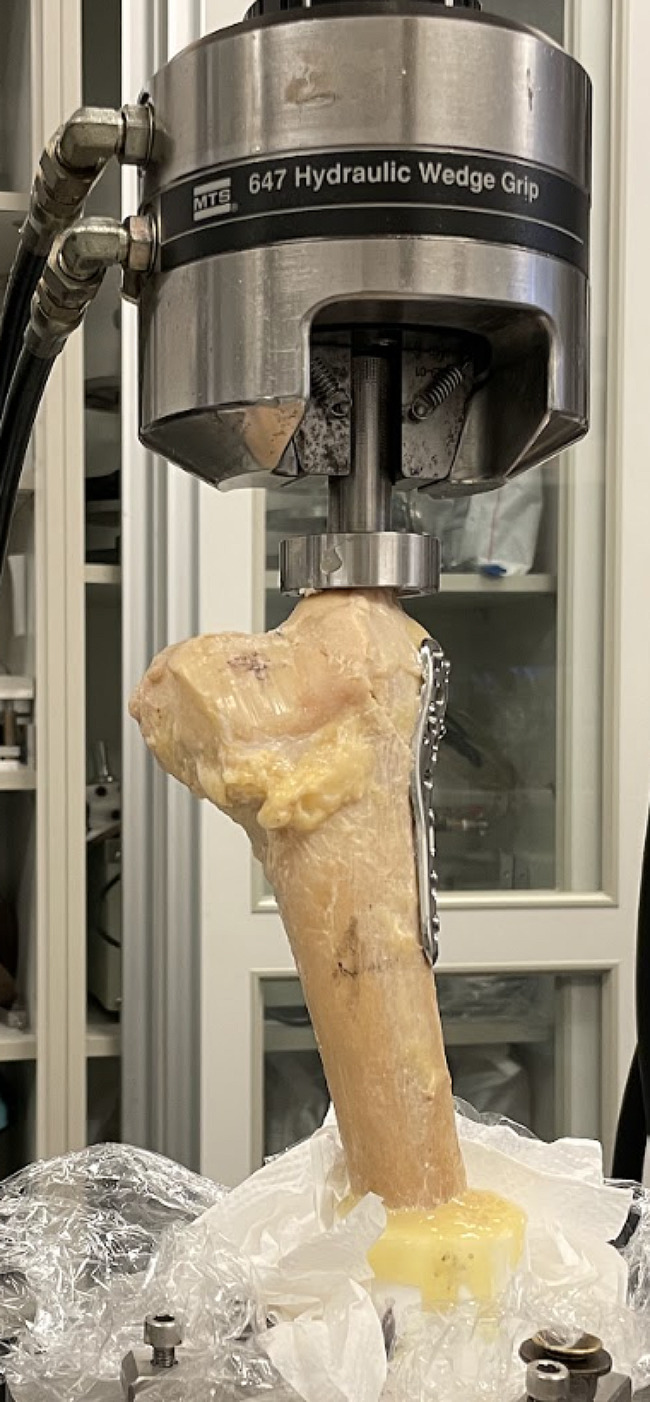
Depicts the set-up of the biomechanical compression test

The femurs shafts were then fixed to an epoxy mold, whereby the medial condyles were axially subjected to a compression load using the Series 647 MTS Hydraulic Wedge Grip at a constant rate of 2 mm/minute until an end point of 20 mm (see Fig. 3). The force across the distal femur was measured at every 0.05 mm intervals to obtain a force-displacement curve. Any fracture of the bone before it reached 20 mm was counted as a failure.

**Fig. 4:**
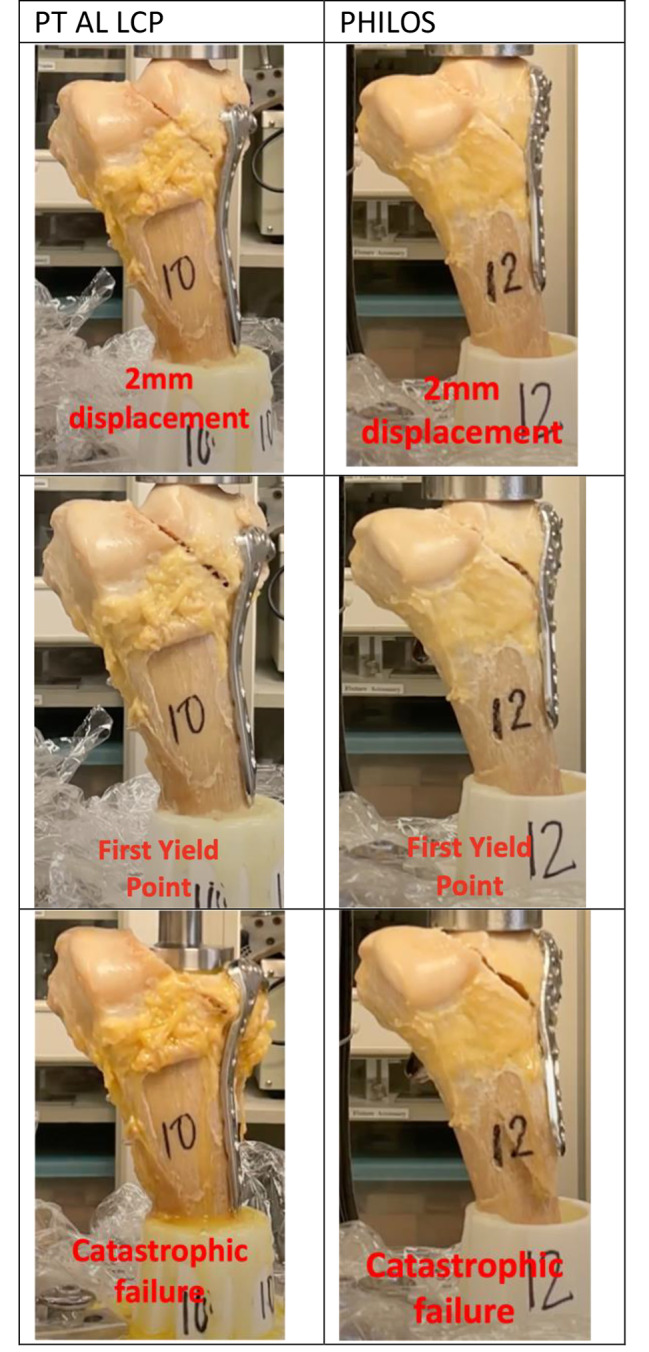
The set-up for mechanical compression test

For this part of the study, the primary outcome was to determine the implant that can sustain the highest force at 2 mm displacement, as the aim of anatomical reduction is to achieve less than 2 mm articular step. The secondary outcomes include the first yield point, the second yield point, the point of catastrophic (ultimate) failure for each implant, measuring both the displacement and the force required at these junctures, and the Young’s modulus for each plate. Figure 4 depicts the serial mechanical compression for each plate.

The first yield point was defined as the point where the force-displacement graph loses its proportionality. The second yield point was the second point where the force-displacement graph loses its proportionality. The point of catastrophic failure was defined as the point at which beyond, there would be implant failure with complete fracture. The mode of failure at yield was noted for every specimen and described qualitatively.

### Statistical analysis

For the first measurement part, a Kruskal Wallis test was used. For the second mechanical part, a paired T-test was used to look for any significant difference between the two plates in terms of forces it could sustain prior to failure. All statistical analysis was done with the SPSS (Statistical Package for the Social Sciences) version 28.

## Results

### Best anatomical fit

**Table 1:** Outcomes of best fit per plate

Outcome	Place	Plate	Result	*p* value (intra-rank)	*p* value (inter-rank)
Metaphysis offset (mm)	1st	PT AL LCP	0.6	-	*p* < 0.001
	2nd	PT M LCP	3.17	-	*p* = 0.004
	3rd	PHILOS, DT M LCP	5.08, 8.09	*p* = n.s.	-
Distal offset (mm)	1st	PT AL LCP, PT M LCP, PHILOS	0.0, 0.9, 1.1	*p* = n.s.	-
	2nd	DT M LCP	30.3	-	*p* = 0.001
Distal distance (mm)	1st	PT AL LCP	20.6	-	*p* = 0.02
	2nd	DT M LCP	23.6	-	*p* = 003
	3rd	PHILOS	26.1	-	*p* = 0.02
	4th	PT M LCP	29.3	-	-
Hoffa screws	1st	PT AL LCP, PHILOS	2.28, 2.41	*p* = n.s.	*p* < 0.001
	2nd	DT M LCP, PT M LCP	1.41, 1.66	*p* = n.s.	
Condyle screws	1st	DT M LCP, PHILOS	3.56, 3.91	*p* = n.s.	*p* < 0.001
	2nd	PT M LCP, PT AL LCP	1.59, 1.61	*p* = n.s.	
Joint penetration screws	1st	PT AL LCP, DT M LCP, PT M LCP	0	*p* = n.s.	-
	2nd	PHILOS	1.09	-	*p* < 0.001

The results of anatomical fit were shown in Table 1. In terms of the outcomes focused on plate placement (metaphysis offset, distal offset, and distal distance), the PT AL LCP is the only plate that ranks top in all three categories. In terms of the number of screws placed in the distal femur, the PHILOS plate is the only plate that ranks top in both screws in the Hoffa fragment and the medial femoral condyle. However, the PHILOS is also the only plate that gives rise to screws penetrating the joint surface.

From the best fit analysis, the PT AL LCP was the plate with the best anatomical fit, ranking top in 5 categories. The PHILOS plate came second, ranking top in 3 categories. The DT M LCP and the PT M LCP were tied for third, with both plates having only top scores in 2 categories. Therefore, the PT AL LCP and the PHILOS plate were chosen for the biomechanical study.

### Biomechanical results

**Table 2:** Results of the biomechanical results

Letter	Outcome	PT AL LCP	PHILOS	*p* value
A	Force at 2 mm displacement (N)	889	947	*p* = 0.39
B	First yield force (N)	1927	2188	*p* = 0.26
	First yield displacement (mm)	5.83	5.79	*p* = 0.47
C	Second yield force (N)	1738	1925	*p* = 0.31
	Second yield displacement (mm)	5.88	5.37	*p* = 0.34
D	Catastrophic failure force (N)	3321	2815	*p* = 0.18
	Catastrophic failure displacement (mm)	24.4	17.5	*p* = 0.004
E	Young’s modulus (N/mm^2^)	476	440	*p* = 0.37

**Fig. 5:**
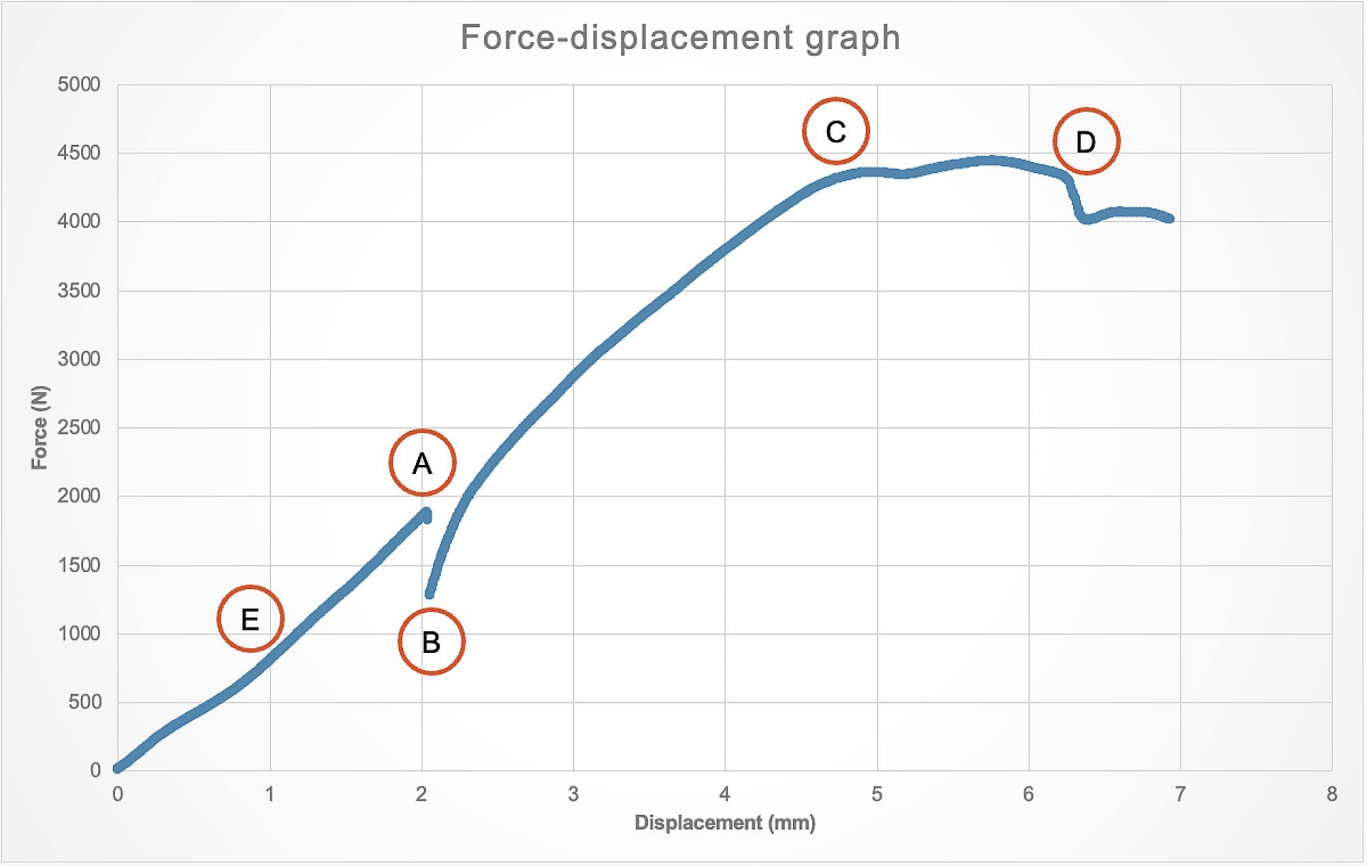
Shows an example of the force-displacement graph; **A** represents the force at 2 mm displacement. **B** represents the first yield point. **C** represents the second yield point. **D** represents catastrophic failure. **E** represents the Young’s modulus

The results of the biomechanical tests were shown in Table 2. With regards to our primary outcome, the force needed to create 2 mm displacement for the PT AL LCP was 889 N whilst the force for PHILOS was 947 N (*p* = 0.39). No specimen failed before 2 mm displacement. For the secondary outcomes, the PT AL LCP proved superior to the PHILOS in terms of the displacement at catastrophic failure, with the PT AL LCP failing at 24.4 mm compared with the PHILOS at 17.4 mm (*p* = 0.004). However, the forces sustained at catastrophic failure between the two plates were not statistically significant (PT AL LCP 3321 N, PHILOS 2815 N; *p* = 0.184). There were no statistical differences between the two plates at the first and second yield point and the Young’ modulus (see Appendix 1). The mode of failure at yield point for all cadavers was a compression fracture of the medial condyle (see Appendix 2). A force-displacement graph (Fig. 5) was obtained for each plate.

## Discussion

The aim for any distal femur fracture fixation is for an anatomical reduction of the articular surface followed by a stable internal fixation [7]. Although the articular surface can be fixed with lag screws, if the fracture extends to the metaphyseal region, a buttress plate is necessary to counteract the vertical shear forces. This has brought on the development of the lateral femoral condyle locking plate [8]. However, in comminuted distal femur fractures, there are high rates of varus collapse with implant failure with using a single lateral plate. This is primarily due to unopposed force of the adductor magnus over the medial side [9, 10]. Hence, there has been increasing evidence for double fixation methods when fixing a comminuted distal femur fracture [11, 12].

Popular options of double fixation of a comminuted distal femur fracture include a dual plating construct or a nail-and-plate construct [13, 14]. Stoffel reviewed multiple studies in 2022 [15]. Although there was no statistical analysis, he concluded that both constructs gave high union rates and satisfactory functional outcomes; choosing which constructs to use relied on balancing the pros and cons with each other. A dual plating construct gave more rigidity, but at the cost of soft tissue stripping. On the other hand, a nail-and-plate construct allowed for immediate post-operative weightbearing as the retrograde intra-medullary nail serves as a load sharing device but cannot be used in periprosthetic fractures. Another systematic review in 2021 concluded that dual plating system led to faster fracture healing rates at the cost of a longer surgical duration, but no difference in non-union rates [16].

As there are no commercially available anatomical plates for the medial condyle, many substitutes have been used but there has been no consensus to date to the best available option. Use of a proximal tibia LCP to fix a medial femoral condyle fracture was first reported in 2020 [3]. The authors reported good functional (ROM 0-120°) and radiographical outcome after fixation of a medial femoral condyle fracture (AO classification 33-B2) using a proximal tibia LCP. Another case report described fixation of a comminuted medial femoral condyle fracture using a D-shaped calcaneal plate with a good functional outcome (ROM 0-110°) afterwards [4]. Another option would be to use the reconstruction plate. Although there has been no reported literature for fixation of the medial femoral condyle using the reconstruction plate, we postulated that contouring of the reconstruction plate would be needed to fit along the medial condyle, which would decrease the strength of the plate.

In our study, we used a straight non-contoured plate to fix the medial femoral condyle. Hohenberger described in 2021 a novel technique to fix distal femur fractures by placing a pre-bent medial helical plate, which would surround the bone spirally up to 180° from medial to lateral [17]. Biomechanical studies have shown that this medial helical plate is superior to straight lateral plates in terms of resistance to varus deformation and higher endurance to failure in comminuted fractures [18]. Further biomechanical studies of the medial helical plate in medial femoral condyle split fractures would be helpful.

In clinical practice, it is not uncommon to encounter comminuted distal femur fractures with Hoffa fragments requiring open reduction internal fixation. It is important therefore, to choose plates which give us good screw fixation into the Hoffa fragment. Conventional straight or reconstruction plates only allows limited number of screws for the fixation of the Hoffa fragment [19]. On the other hand, anatomically pre-contoured lateral distal femur plates are designed for a more anterior placement, and hence do not provide sufficient fixation for Hoffa fragments. From our study, both the PT AL LCP and the PHILOS plate were able to give at least 2 screws into the Hoffa fragment, and thus should be chosen for fixation of the Hoffa fragment.

In our biomechanical study, we found no statistical difference for the force measured at 2 mm displacement (*p* = 0.39). In both plates, the first yield point came out to be 1927 N and 2188 N for the PT-AL LCP and the PHILOS plate respectively, which is similar to other biomechanical studies regarding the distal femur. El-Zayat et al. reported failure of 5 out of 7 cadaveric specimens in his biomechanical distal femur study before 2000 N [20]. Regarding the mode of failure at yield point, all cadavers failed due to fracturing of the medial condyle. This may be because the limiting factor is the bone mineral density, rather than the choice of implant. This suggests that both plates are suitable for fixation, as the bone gives way before the plate does, which may explain why the primary outcome of the study is statistically insignificant. To make it a fair test, we applied 3 proximal locking screws and 4 distal locking screws for both the PT AL LCP and the PHILOS. Even though the PHILOS plate can allow up to 9 screws to be applied distally, we expect there would be no difference because the common failure mechanism is initiated through fracture or subchondral bony collapse distal to the implant instead of screw hole failure or plate deformation. (Appendix 1). Also, all cadavers were fixed using 3.5 mm screws. Although using 4.5 mm screws would have given an even more stable construct, there was concern about overcrowding of the screws leading to interference of the screw trajectory, and our study concluded that a fixation using 3.5 mm screws was stable enough as the first mode of failure is the subchondral collapse of the femoral condyle.

The PT AL LCP was able to sustain 24 mm of displacement before failure (*p* = 0.004) versus 17 mm of displacement for the PHILOS. This may be because of the design of the PT AL LCP being naturally longer in the metaphyseal area and thus giving better buttress effect (in our study, the 10-hole PT AL LCP was used VS the standard 3 shaft hole PHILOS plate). However, this end point represents unacceptable varus deformity and is already far beyond the generally accepted clinically relevant threshold of failure.

In the design of this study, only mechanical aspects have been considered, whereas in real-life trauma situations, management of the soft tissue status should take precedence and one should follow the slogan span-scan-plan. Traditionally, the quality of the CT images have been affected by scattering caused by the presence of the fixator pins. Recent studies have suggested that MRI scans offer good analysis of the fracture patterns around the knee despite presence of the fixator pins [21, 22].

This study has a few limitations. Firstly, the cadaveric specimens were subjected to a single axial compression load only, whereas the knee is normally subjected to a multitude of load directions. Secondly, the cadaveric specimens were embalmed; formaldehyde stiffens the tissue and may change the biomechanical properties of the bone. Next, these plates are applied to cadavers with an isolated medial condyle fracture; we have no biomechanical data for comminuted distal femur fractures or dual plating system. Finally, the variation in BMD in each cadaver may increase the variation of the results of the study but a randomized design should have limited this effect.

## Conclusion

While there remains no clear-cut winner as the best substitute for an anatomically shaped medial femoral condyle locking plate, our study suggested that specific locking plates for the medial condyle may not be needed. Both the commonly used anterolateral proximal tibia locking compression plate and the proximal humerus locking plate would be good alternatives. Between the two, we would recommend the anterolateral proximal tibia plate as the superior option because of the ability to put in maximal number of Hoffa screws without any screws penetrating the articular surface.

### Electronic supplementary material

Below is the link to the electronic supplementary material.

Supplementary Material 1

## Data Availability

The datasets generated and/or analysed during the current study are not publicly available due to new data generated from own study but are available from the corresponding author on reasonable request.

## Declarations

### Cadaveric source

We purchased our cadavers from an American company called “Research For Life” following their standard donation and consent practices.

## Abbreviations

DT M LCP: Distal Tibia Medial Plate
PHILOS: Proximal Humerus Internal Locking System
PT AL LCP: Proximal Tibia Anterolateral Plate
PT M LCP: Proximal Tibia Medial Plate
SPSS: Statistical Package for the Social Sciences
