# Supplementary material for: Determination of the ideal plate for medial femoral condyle fracture fixation: an anatomical fit and biomechanical study
Source: BMC Musculoskelet Disord. 2024 Apr 16;25:296. doi: 10.1186/s12891-024-07374-5 (PMC11020340; doi:10.1186/s12891-024-07374-5)
Supplement: Supplementary file 1 — Supplementary Material 1 [file 12891_2024_7374_MOESM1_ESM.docx]

**Additional Materials**

*File name:* Additional file 1

*File format:* .docx

*Title of data:* Appendix

*Description of data:* Appendix table of data on 1^st^ yield point force and displacement and photographs on failure at yield point.

**Appendix**

Appendix 1 (First yield point force and displacement)

| Specimen number | Plate | Yield force (N) | Yield displacement (mm) |
| --- | --- | --- | --- |
| 1 | PHILOS | 4447 | 5.76 |
| 2 | PT AL LCP | 2021 | 25.31 |
| 3 | PHILOS | 2380 | 5.83 |
| 4 | PHILOS | 958 | 6.07 |
| 5 | PT AL LCP | 2351 | 5.59 |
| 6 | PT AL LCP | 1012 | 6.41 |
| 7 | PT AL LCP | 2361 | 5.08 |
| 8 | PT AL LCP | 1228 | 3.69 |
| 9 | PT AL LCP | 945 | 3.09 |
| 10 | PT AL LCP | 1370 | 2.84 |
| 11 | PT AL LCP | 3125 | 4.96 |
| 12 | PHILOS | 731 | 2.76 |
| 13 | PT AL LCP | 3167 | 4.93 |
| 14 | PHILOS | 2224 | 4.04 |
| 15 | PHILOS | 1606 | 6.25 |
| 16 | PHILOS | 2111 | 6.85 |
| 17 | PHILOS | 3237 | 3.92 |
| 18 | PT AL LCP | 2527 | 4.45 |
| 19 | PHILOS | 4065 | 9.25 |
| 20 | PHILOS | 554 | 9.46 |
| 21 | PT AL LCP | 3545 | 5.02 |
| 22 | PHILOS | 1267 | 5.32 |
| 23 | PT AL LCP | 1447 | 3.32 |
| 24 | PT AL LCP | 966 | 3.84 |
| 25 | PHILOS | 1553 | 5.03 |
| 26 | PHILOS | 3050 | 5.31 |
| 27 | PT AL LCP | 916 | 3.16 |
| 28 | PHILOS | 2447 | 5.20 |

Appendix 2 (Failure at yield point)

| 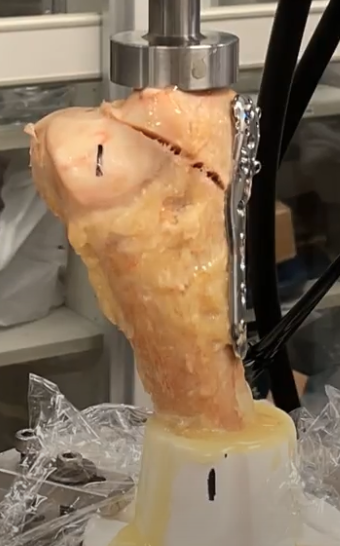 | 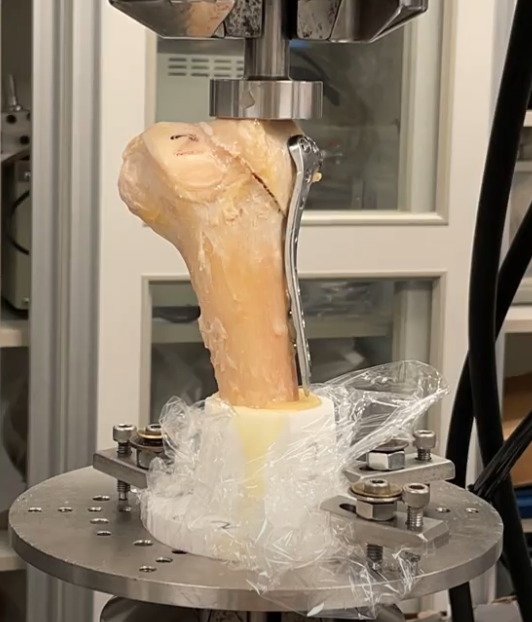 | 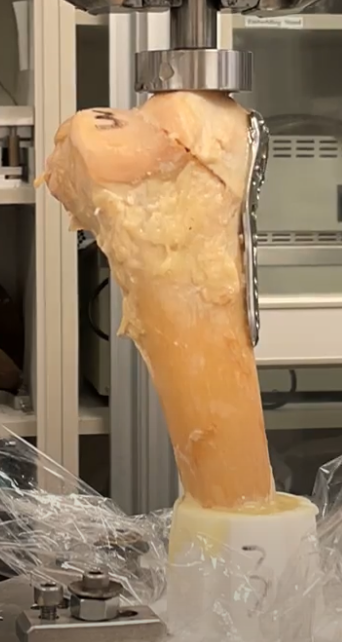 | 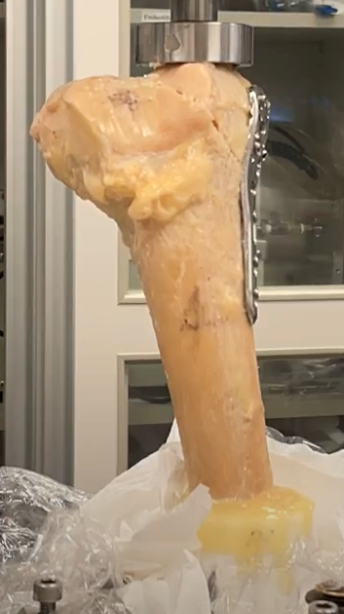 | 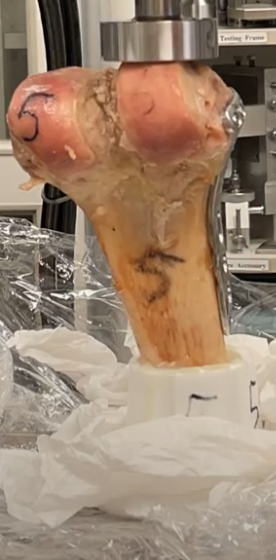 | 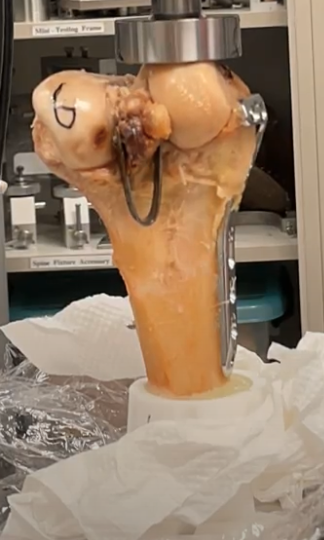 | 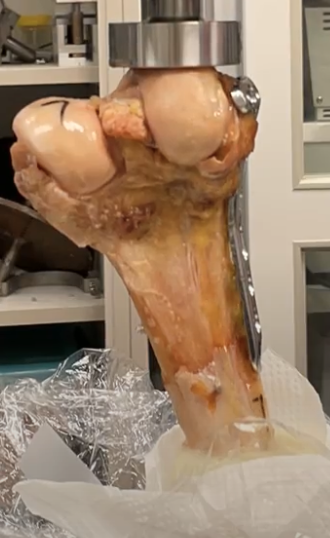 |
| --- | --- | --- | --- | --- | --- | --- |
| 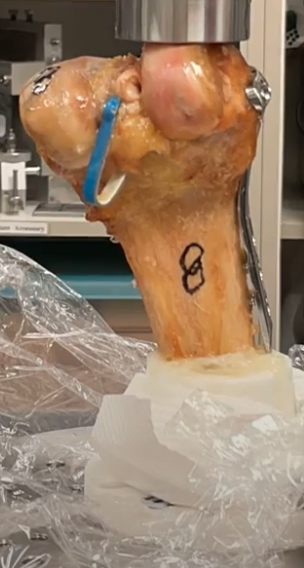 | 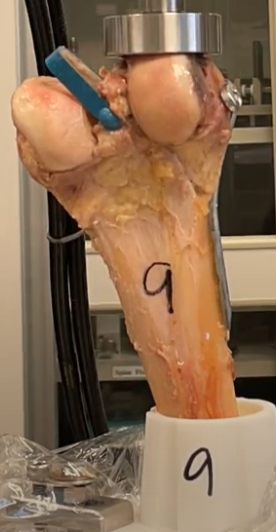 | 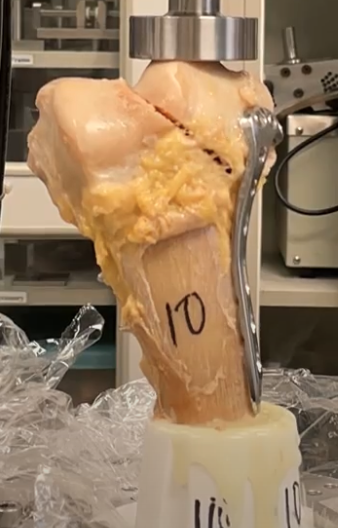 | 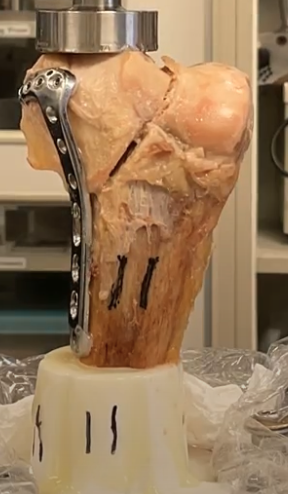 | 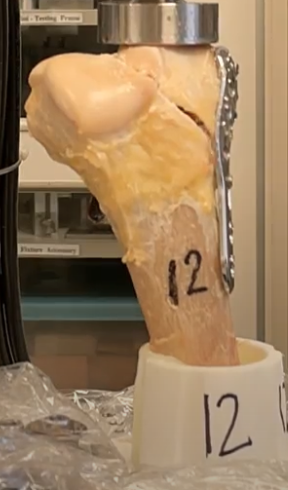 | 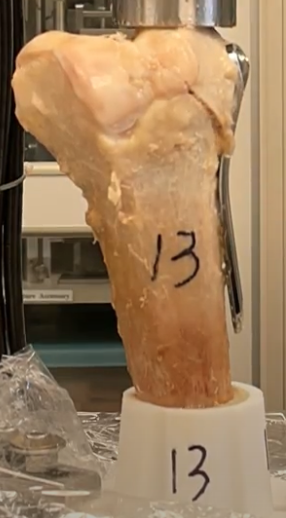 | 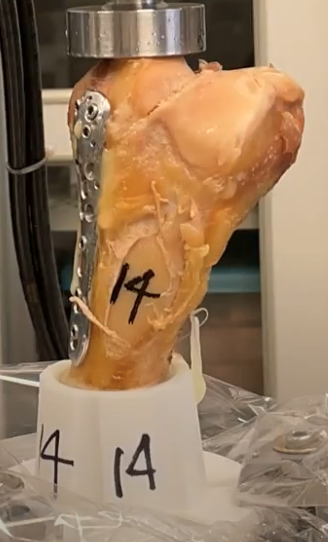 |
| 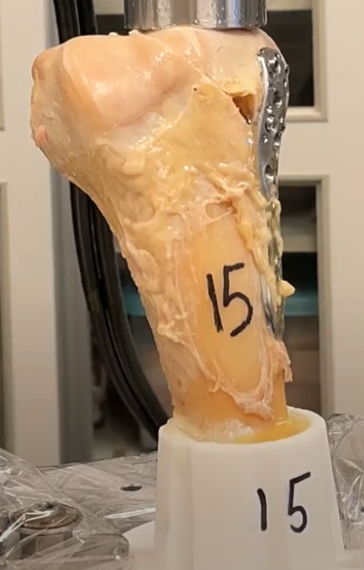 | 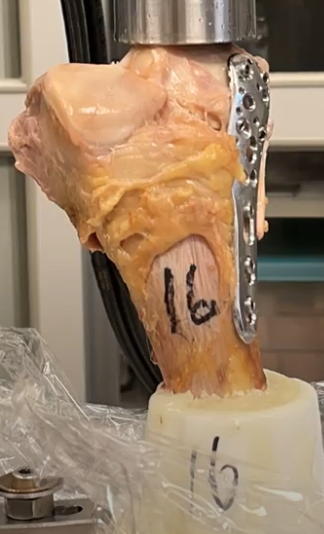 | 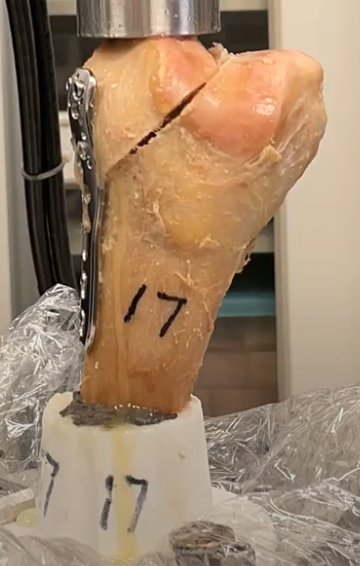 | 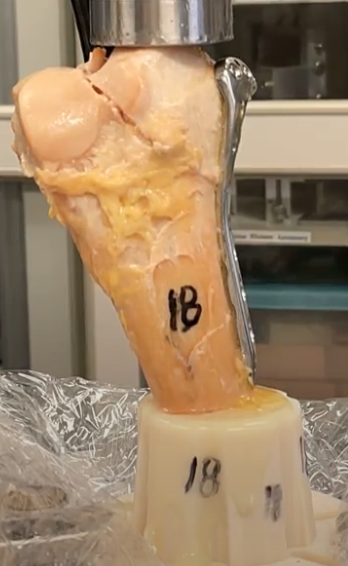 | 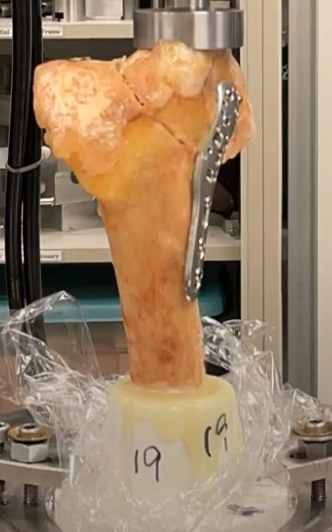 | 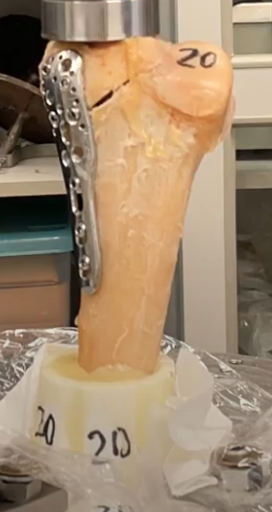 | 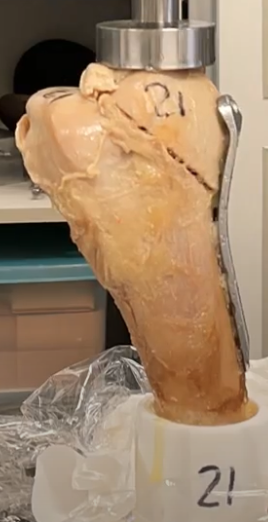 |
| 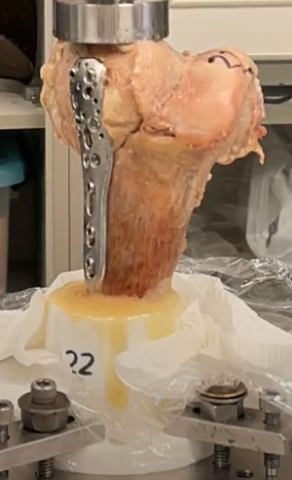 | 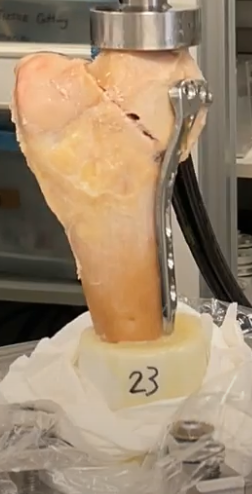 | 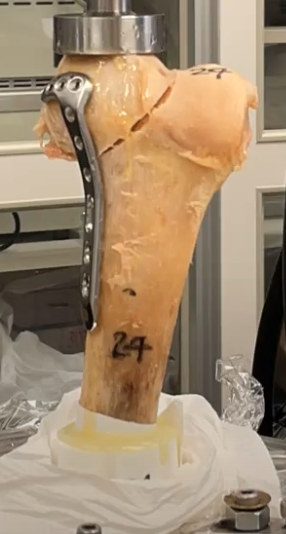 | 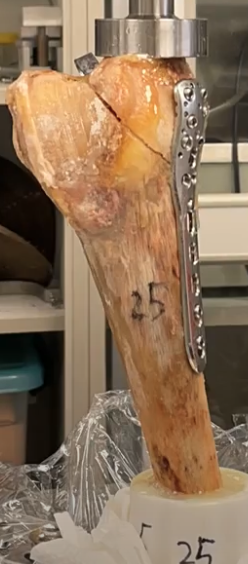 | 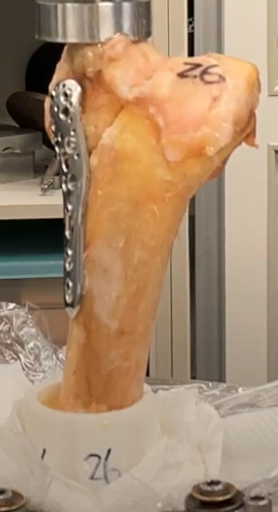 | 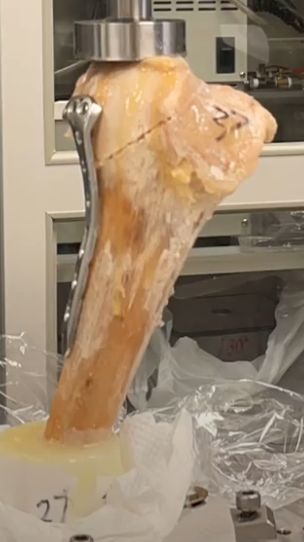 | 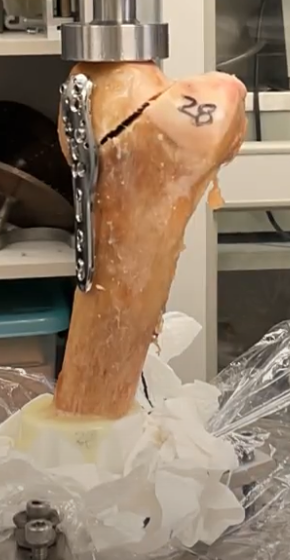 |
